# Trends and Predictors of Total Neoadjuvant Therapy in Rectal Cancer: A Bi‐National Registry Study Across Australia and New Zealand

**DOI:** 10.1111/ans.70218

**Published:** 2025-06-10

**Authors:** Ishraq Murshed, Sergei Bedrikovetski, Zachary Bunjo, Hidde M. Kroon, Michelle Thomas, Tarik Sammour

**Affiliations:** ^1^ Discipline of Surgery, Adelaide Medical School, Faculty of Health and Medical Sciences Royal Adelaide Hospital Adelaide Australia; ^2^ Colorectal Unit, Department of Surgery University of Adelaide Adelaide South Australia Australia

**Keywords:** chemoradiotherapy, chemotherapy, locally advanced rectal cancer, total neoadjuvant therapy

## Abstract

**Background:**

Total Neoadjuvant Therapy (TNT) has significantly improved outcomes in locally advanced rectal cancer. Despite rising use and inclusion in international guidelines, adoption patterns in Australia and New Zealand (ANZ) remain unclear. This study determines bi‐national patterns and predictors of TNT utilisation over a 6‐year period.

**Methods:**

A retrospective population‐based bi‐national registry cohort study analysed data from the ANZ Bowel Cancer Outcome Registry from 2018 to 2024. Patients diagnosed with primary locally advanced rectal cancer (LARC) and treated with standard neoadjuvant therapy (SNT), consisting of long‐course chemoradiotherapy or short‐course radiotherapy, or TNT were included. The primary outcome was the incidence of TNT utilisation over time and determining pre‐treatment predictive factors.

**Results:**

Of 33 270 patient entries, 3234 eligible LARC patients were identified, with 706 (21.8%) receiving TNT and 2528 (78.2%) receiving SNT. TNT usage increased from 11% in 2018 to 41% in 2023. On multivariable logistic regression, independent factors associated with decreased TNT use were older age (OR: 0.978, 95% CI: 0.970–0.985, *p* < 0.001), absence of multidisciplinary team discussion (OR: 0.113, 95% CI: 0.027–0.472, *p* = 0.003) and private health insurance (OR: 0.619, 95% CI: 0.441–0.869, *p* = 0.006). Factors associated with increased TNT use were clinical T4 tumours (OR: 2.020, 95% CI: 1.245–3.280, *p* = 0.004), node‐positive status (OR: 1.481, 95% CI: 1.118–1.964, *p* = 0.006) and diagnosis during the years 2019–2023 (*p* < 0.05 for all).

**Conclusions:**

TNT is increasingly used in surgical rectal cancer patients captured in the BCOR in ANZ. This study provides a baseline for future benchmarking patterns of rectal cancer management.

## Graphical abstract

Neoadjuvant therapy type for LARC from 2018 to 2023.

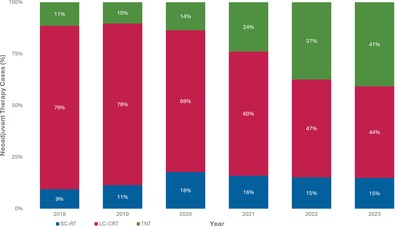

## 1 Introduction

Traditionally, locally advanced rectal cancer (LARC) has been managed with either neoadjuvant long‐course chemoradiotherapy (LC‐CRT) or short‐course radiotherapy (SC‐RT), followed by Total Mesorectal Excision (TME) and selective adjuvant chemotherapy [1]. Although adjuvant chemotherapy improves disease‐free survival (DFS) [2, 3, 4], many patients fail to complete treatment due to poor compliance, toxicity and postoperative complications [5, 6].

Total Neoadjuvant Therapy (TNT), which integrates neoadjuvant (chemo) radiotherapy with upfront systemic chemotherapy, has shown better chemotherapy compliance, higher rates of complete pathological response, and improved DFS rates [7, 8, 9]. Additionally, TNT increases the chances of complete clinical response (cCR), facilitating organ preservation through non‐operative management [10, 11, 12]. As a result, TNT is now recommended in many major international guidelines for LARC [1, 13, 14], and its usage has surged in recent years [15, 16].

Despite robust evidence and its international endorsement, Australian rectal cancer guidelines have not yet incorporated TNT as the recommended treatment for LARC [17]. The extent to which TNT is being adopted in Australia and New Zealand (ANZ) remains unclear. This study was conducted to evaluate TNT usage trends and identify pre‐treatment factors influencing its administration in LARC patients across ANZ, using data from the Bowel Cancer Outcomes Registry (BCOR).

## 2 Methods

### 2.1 Study Design and Ethical Approval

This observational cohort study utilised bi‐national registry data from the BCOR and followed the ‘Strengthening the Reporting of Observational Studies in Epidemiology’ (STROBE) guidelines [18]. Approval was granted from the BCOR Operations Committee and the Central Adelaide Local Health Network Human Research Ethics Committee (reference: 13682).

### 2.2 Data Source

The BCOR, formerly the Bi‐National Colorectal Cancer Audit, is a prospective, multi‐institutional clinical quality registry that collects colorectal cancer treatment and outcome data in ANZ. Established in 2007 and endorsed by the Colorectal Surgical Society of Australia and New Zealand, BCOR participation by clinicians and patients is voluntary and data is self‐entered by participating institutions. However, since 2018, it has been mandatory for all accredited colorectal fellowship training centres in ANZ to register their data. It records surgical events and collects data on pre‐treatment demographics, tumour characteristics, treatment methods, complications, outcomes and clinical quality indicators.

### 2.3 Patient Population

Data were extracted for patients diagnosed with rectal cancer between 1 January 2018 and 25 June 2024. Patients diagnosed before 2018 were excluded to minimise reporting bias since participation was not mandatory for colorectal specialised centres before that time [19] and as TNT had not yet been incorporated into international guidelines, such as from the National Comprehensive Cancer Network and the European Society for Medical Oncology [13, 14]. Eligible patients included those diagnosed with clinical Stage II (T3‐4N0M0) or Stage III (T1‐4 *N* + M0) LARC, defined according to the American Joint Committee on Cancer (AJCC) [20]. Eligibility was determined by clinical TNM stages. If clinical T or N stages were undocumented, patients were still included if their overall AJCC stage was recorded as II or III. Patients with AJCC Stage I or IV disease, non‐rectal tumours, recurrent LARC, or missing staging or neoadjuvant therapy (NAT) information were excluded. Non‐rectal tumours were defined as those located proximal to the rectosigmoid junction (greater than 15 cm from the anal verge) or of non‐adenocarcinoma histology. Recurrent LARC was defined as any local tumour regrowth following primary curative treatment, excluding regrowth during non‐operative management, as surgical treatment had not been performed yet.

### 2.4 Outcome Measures

The primary outcome was the incidence of TNT utilisation over time. The utilisation of NAT is reported in the BCOR as either ‘SC‐RT’, ‘LC‐CRT’ or ‘Other’. SC‐RT and LC‐CRT were grouped together as standard neoadjuvant therapy (SNT) for analysis, as no clear difference in outcomes have been established, and both regimens are commonly used in practice [21]. The ‘Other’ category was used to represent TNT as the only other plausible NAT option for LARC given neoadjuvant chemotherapy alone currently remains a relatively novel treatment option [22] and is unlikely to have been utilised widely during the study period. Additionally, the ‘Other’ category has been used to represent neoadjuvant TNT for rectal cancer in the 2022 and 2023 BCOR Annual Reports [19, 23]. Finally, cross‐checking the assumption using the author's local BCOR data yielded a 96% accuracy rate. Trends were analysed up to 2023 to ensure comprehensive coverage of complete reporting years. Data from 2024, though preliminary, were included in the secondary outcome analysis (other than year of diagnosis) to maximise the inclusion of eligible cases. Patients with missing data points were excluded from analysis.

The secondary outcome was identifying pre‐treatment predictive factors associated with TNT utilisation compared to SNT. Pre‐treatment variables included patient‐related factors (age, sex, health insurance status [public or private], American Society of Anaesthesiologists [ASA] physical status, year of diagnosis) and tumour‐related factors (TNM staging and tumour location). Department of Veterans' Affairs insurance was classified as public health insurance.

### 2.5 Statistical Analysis

Continuous variables with a normal distribution were presented as mean (standard deviation [SD]), whereas those with an asymmetrical distribution were reported as median (inter‐quartile range [IQR]). Normality of continuous data was assessed statistically using the Shapiro–Wilk test and graphically with Q‐Q plots. Parametric and non‐parametric continuous variables were compared using the independent *t*‐test or the Mann–Whitney U test, respectively. Categorical variables were presented as frequency (percentage) and compared using the chi‐square test for nominal data and the Mann–Whitney U test for ordinal data. Sample size was determined by the number of cases in the BCOR during the study period. Missing tumour location data was determined from documented height from anal verge when available. A univariate logistic regression (LR) analysis identified significant factors associated with TNT utilisation, with those meeting a *p*‐value < 0.10 included in a multivariable LR analysis to determine independent predictors. Clinical T1 and T2 tumours were grouped, and clinical N stages were grouped as node‐negative (N0) and node‐positive (N1 and N2) for LR. A *p*‐value < 0.05 on multivariable LR was considered statistically significant. Statistical analyses were performed using IBM SPSS Statistics for Windows, version 27 (IBM Corp., Armonk, N.Y., USA).

## 3 Results

**FIGURE 1:**
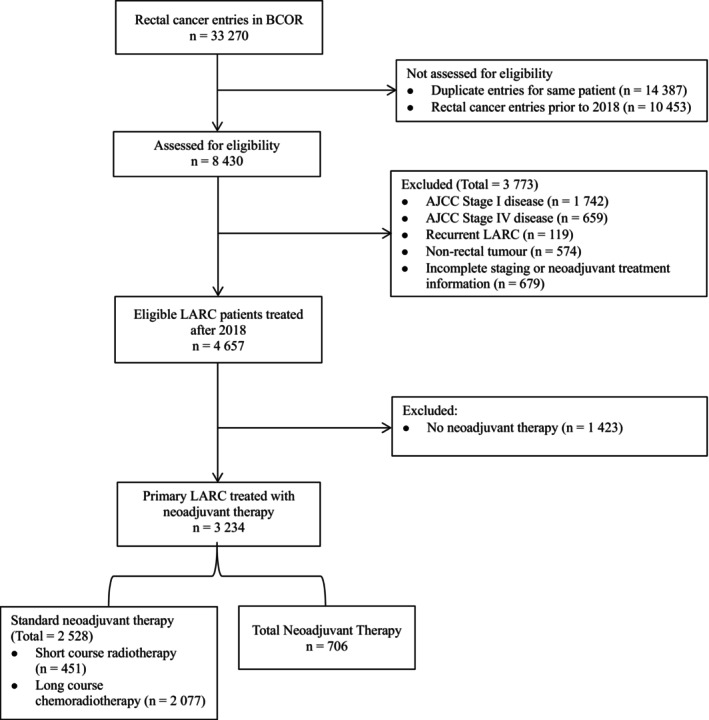
STROBE flow chart.

Out of 33 270 rectal cancer patient entries in the BCOR, 8430 patients were assessed for eligibility after excluding pre‐2018 entries (*n* = 10 453) and duplicates (*n* = 14 387). Among these, 3773 patients were excluded due to AJCC Stage I (*n* = 1742) or Stage IV (*n* = 659) disease, recurrent LARC (*n* = 119), non‐rectal tumours (*n* = 574) and incomplete staging or NAT information (*n* = 679). The final eligible cohort consisted of 4657 LARC patients, of whom 1423 (30.6%) did not receive NAT while 3234 (59.4%) did. Among those receiving NAT, 706 (21.8%) patients received TNT, 2528 (78.2%) patients received SNT (SC‐RT = 451 and LC‐CRT = 2077) (Figure 1).

### 3.1 Baseline Characteristics

Baseline characteristics of the NAT patients are summarised in Table 1. The majority were male (65.3%) with a median age of 64 years. Most patients were ASA II (43.5%) or ASA III (31.8%), publicly insured (84%), discussed in multidisciplinary team (MDT) meetings (95.7%), and had Stage III disease (72.5%). Tumours were predominantly T3 (67.6%) or T4 (17.5%), node‐positive (N1 = 41.7%, N2 = 28.2%), and located in the mid (30.8%) or low (60.6%) rectum. Compared to SNT patients, those receiving TNT were significantly younger (63 vs. 64 years, *p* = 0.02), more likely to have public health insurance (91.8% vs. 81.8%, *p* < 0.001), discussed in MDT meetings (99.6% vs. 94.6%, *p* < 0.001), have higher T and N stages (*p* = 0.001 and *p* < 0.001), respectively, and more likely to have upper rectal tumours (*p* < 0.001). Gender, ASA class, and AJCC stage were similar between groups.

**TABLE 1:** Patient demographics.

Characteristic	Standard neoadjuvant therapy *N* = 2528	Total neoadjuvant therapy *N* = 706	*p*
Sex (%)			0.535
Male	1644 (65.0)	468 (66.3)	
Female	884 (35.0)	238 (33.7)	
Age in years, median (IQR)	64 (55–73)	63 (53–72)	0.018
ASA score (%)			0.158
I	238 (9.4)	48 (6.8)	
II	1156 (45.7)	252 (35.7)	
III	822 (32.5)	207 (29.3)	
IV	43 (1.7)	8 (1.1)	
V	1 (0.0)	0 (0.0)	
Not reported	268 (10.6)	191 (27.1)	
Insurance type (%)			< 0.001
Public	2068 (81.8)	648 (91.8)	
Private	456 (18.0)	58 (8.2)	
Not reported	4 (0.2)	0 (0.0)	
MDT discussion (%)			< 0.001
Yes	2392 (94.6)	703 (99.6)	
No	121 (4.8)	2 (0.3)	
Not reported	15 (0.6)	1 (0.1)	
Stage (%)			0.133
Stage II	608 (24.0)	134 (19.0)	
Stage III	1867 (73.9)	484 (68.5)	
Not reported	53 (2.1)	88 (12.5)	
Clinical T stage (%)			0.001
1	11 (0.4)	0 (0.0)	
2	172 (6.8)	29 (4.1)	
3	1823 (72.1)	363 (51.4)	
4	441 (17.4)	124 (17.6)	
Not reported	81 (3.2)	190 (26.9)	
Clinical N stage (%)			< 0.001
0	559 (22.1)	84 (11.9)	
1	1137 (45.0)	211 (29.9)	
2	698 (27.6)	215 (30.4)	
Not reported	134 (5.3)	196 (27.8)	
Tumour height from anal verge (%)			< 0.001
Upper rectum > 12 cm	146 (5.8)	94 (13.3)	
Middle rectum 8‐12 cm	766 (30.3)	229 (32.4)	
Low rectum < 8 cm	1606 (63.5)	355 (50.3)	
Not reported	10 (0.4)	28 (4.0)	

### 3.2 Neoadjuvant Therapy Utilisation

**FIGURE 2:**
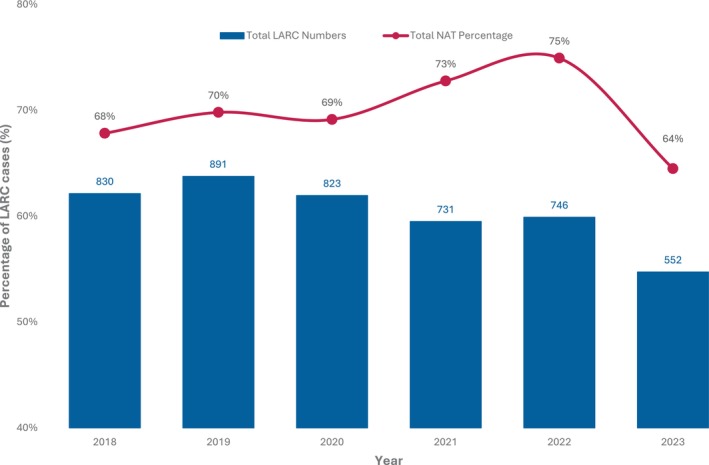
Total number of LARC and neoadjuvant therapy rates: 2018–2023.

**FIGURE 3:**
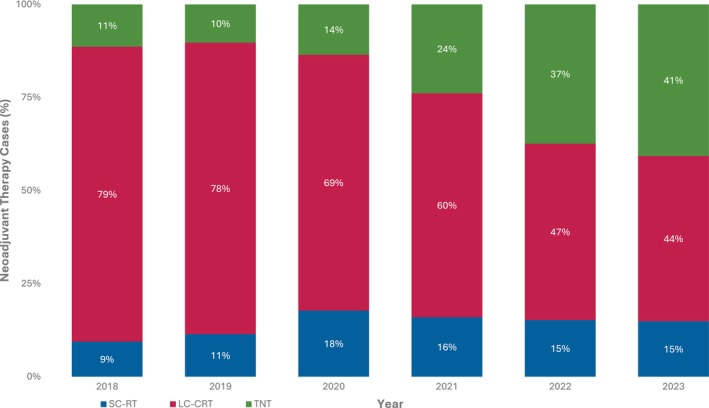
Neoadjuvant therapy types: 2018–2023.

NAT utilisation rose from 68% in 2018 to 75% in 2022, then declined to 64% in 2023, coinciding with the lowest annual LARC cases recorded for that year (552 entries) (Figure 2). TNT utilisation as a proportion of NAT increased from 11% (64 cases) in 2018 to 41% (145 cases) in 2023. During the same period, SC‐RT utilisation increased slightly from 9% to 15%, whilst LC‐CRT rates significantly decreased from 79% to 44% (Figure 3).

### 3.3 Factors Associated With TNT Utilisation

Table 2 displays the complete results of LR. Univariate LR analysis identified several factors associated with TNT utilisation, including age (*p* = 0.02), insurance type (*p* < 0.001), MDT discussion (< 0.001), clinical T stage (*p* < 0.001), clinical N stage (*p* < 0.001), tumour height from anal verge (*p* < 0.001) and year of diagnosis (*p* < 0.001). Multivariable LR analysis revealed that older age (OR: 0.978 per year, 95% CI: 0.970–0.985, *p* < 0.001), private insurance (OR: 0.619, 95% CI: 0.441–0.869, *p* = 0.006), and the absence of MDT discussion (OR: 0.113, 95% CI: 0.027–0.472, *p* = 0.003) were associated with a lower likelihood of TNT utilisation. Clinical T4 stage (OR: 2.020, 95% CI: 1.245–3.280, *p* = 0.004), clinical node‐positive status (OR: 1.481, 95% CI: 1.118–1.964, *p* = 0.006), and diagnosis in any years from 2019 onwards (*p* < 0.05 for all years) were all associated with a higher likelihood of TNT utilisation. Although TNT utilisation increased substantially for mid‐low rectal cancers from 2018 to 2024, tumour height was not significantly associated with TNT receipt on multivariable LR, suggesting other factors contributed to the increase in incidence seen.

**TABLE 2:** Factors associated with TNT usage compared to standard neoadjuvant therapy.

Factors		Univariate analysis		Multivariable analysis	
		Odds ratio (95% CI)	*p*	Odds ratio (95% CI)	*p*
Sex	Male	Reference	0.535		
	Female	0.946 (0.793–1.128)			
Age		Reference	0.022	Reference	< 0.001
	Increasing age (years)	0.993 (0.987–0.999)		0.978 (0.970–0.985)	
ASA class*	I	Reference	0.403		
	II	1.081 (0.770–1.517)			
	III	1.249 (0.884–1.764)			
	IV	0.922 (0.408–2.086)			
Insurance type	Public	Reference	< 0.001	Reference	0.006
	Private	0.424 (0.320–0.563)		0.619 (0.441–0.869)	
MDT discussion	Yes	Reference	< 0.001	Reference	0.003
	No	0.056 (0.014–0.228)		0.113 (0.027–0.472)	
Clinical T stage	1–2	Reference	0.004	Reference	
	3	1.257 (0.836–1.889)		1.456 (0.933–2.272)	0.098
	4	1.774 (1.143–2.754)		2.020 (1.245–3.280)	0.004
Clinical N stage	Negative	Reference	0.001	Reference	0.006
	Positive	1.545 (1.200–1.989)		1.481 (1.118–1.964)	
Tumour height from anal verge	Upper rectum (> 12 cm)	Reference	< 0.001	Reference	
	Middle rectum (8–12 cm)	0.464 (0.345–0.626)		0.975 (0.620–1.532)	0.912
	Low rectum (< 8 cm)	0.343 (0.259–0.456)		0.965 (0.627–1.485)	0.870
Year of diagnosis	2018	Reference	< 0.001	Reference	
	2019	1.118 (0.776–1.611)		1.941 (1.051–3.585)	0.034
	2020	1.377 (0.961–1.973)		3.008 (1.675–5.401)	< 0.001
	2021	2.624 (1.875–3.671)		6.688 (3.844–11.637)	< 0.001
	2022	5.062 (3.677–6.968)		16.827 (9.843–28.767)	< 0.001
	2023	5.802 (4.118–8.175)		20.091 (11.578–34.865)	< 0.001

* 1 patient with ASA class V was excluded from analysis as the probability could not be computed.

## 4 Discussion

This study represents the first bi‐national evaluation of TNT utilisation for LARC in ANZ. Analyzing data from the prospectively maintained BCOR database, there was a consistent and significant increase in TNT utilisation over time. Although overall rates of NAT declined during this period, TNT utilisation as a proportion of NAT rose significantly from 11% (64 cases) in 2018 to 41% (145 cases) in 2023. The trend corresponded with a complementary decrease in LC‐CRT, suggesting a shift towards TNT. Rates of SC‐RT remained stable, rising slightly from 9% in 2018 to 15% in 2023. Multivariable LR analysis identified pre‐treatment factors associated with increased TNT utilisation included clinical T4 stage, node‐positive status, and more recent years of diagnosis. Conversely, increased age, private health insurance, and the absence of MDT discussion were associated with decreased TNT utilisation.

Although few studies have focused on the adoption of TNT in LARC, our findings align with global trends [15, 16]. Using the National Cancer Database (NCDB), Liu, et al. reported TNT utilisation in the US rose from 6.1% in 2016 to 34.6% in 2020 [15], whereas Unuvar et al. estimated an increase from 57.6% in 2013 to 68.5% in 2022 [16]. A challenge in interpreting NCDB data is that both LC‐CRT and TNT are coded as receiving similar sequences of NAT, complicating distinctions between the two. Given that phase III data on TNT was still emerging in the 2010s, a baseline TNT rate exceeding 50% is likely to be inaccurate and the more conservative estimate by Lui et al. may better reflect TNT adoption in the US. The BCOR database, however, does differentiate between SC‐RT, LC‐CRT, and ‘other’ NAT, facilitating a more distinctive analysis. Recent BCOR analysis indicates that up to 17% of all rectal cancers patients in ANZ receive TNT [19]. As current international guidelines recommend TNT specifically for LARC, our analysis provides more clinically relevant insights into treatment trends in ANZ, demonstrating that, despite the absence of local guideline endorsement, LARC management in ANZ has kept pace with international recommendations.

Our analysis identified several independent factors influencing TNT utilisation. Older age, private health insurance, and absence of MDT discussion were all associated with lower likelihoods of receiving TNT. Although some studies have shown mixed results regarding age and TNT receipt [15, 16], our findings are in line with other reports indicating reduced initiation of combined therapy in elderly patients [24], due to poorer tolerance [25], higher risk of adverse events [26, 27] and higher rates of treatment discontinuation [25]. Despite these challenges, older patients who do receive optimal therapy have similar disease‐related outcomes to younger patients [28, 29, 30]. Concerns about adverse events may lead clinicians to prefer SNT for older patients. However, because treatment sequence affects adverse event profiles [31], and some TNT patients may achieve a cCR and avoid surgery altogether, shared decision‐making is essential. Pre‐operative MDT discussions, which are endorsed by clinical practice guidelines [17, 32], are associated with more effective treatments resulting in better outcomes for rectal cancer patients [33, 34]. As TNT involves coordination between multiple specialties, the absence of a pre‐operative MDT discussion would decrease the likelihood of TNT receipt.

Interestingly, our study found that private health insurance was associated with a lower likelihood of TNT receipt. In contrast to the US, where lack of private health insurance is linked with healthcare disparities and worse cancer‐related outcomes [35], ANZ has a universal public healthcare system. Major hospitals in ANZ, including most university‐affiliated and academic tertiary hospitals, are public hospitals. In Australia, patients with private insurance were more likely to receive colorectal cancer surgery [36], although the impact on cancer‐related outcomes has been inconsistent [36, 37]. Given that 82% of BCOR cases have been reported from public hospitals [19], it is plausible that emerging treatments such as TNT are more quickly adopted in these settings compared to private practice.

We observed that higher clinical T and N stages were associated with a higher likelihood of receiving TNT. Compared to T1‐2 tumours, T4 tumours had double the odds of receiving TNT, whereas node‐positive tumours had 50% higher odds. T3 tumours showed a trend towards greater odds of TNT receipt, but this did not reach statistical significance. Lui, et al. found T3N0 tumours were less likely to receive TNT, whereas T3N+, T4N0 and T4N+ were more likely to receive it [15]. Similarly, Unuvar et al. found that AJCC Stage III LARC, compared to Stage II, were more likely to receive TNT [16]. Landmark trials, such as RAPIDO and PRODIGE‐23, included few node‐negative patients (8% and 10%, respectively) [8, 9], and the role of chemotherapy in node‐negative LARC remains a topic of debate [2]. These factors likely explain the preference for TNT in node‐positive LARC to achieve earlier systemic control, as evidenced by the higher rates of TNT adoption for node‐positive T3 and T4 tumours compared to their node‐negative counterparts.

This study has several limitations. First, registry‐based analyses are at risk of incomplete data entry and reporting bias. Although the BCOR is the largest colorectal cancer registry in ANZ, it only captures 20%–30% of annual cases [19]. Given the BCOR only captures a small proportion of patients across ANZ and only includes patients who have had surgery, this constrains the accuracy and applicability of our results to all patients diagnosed with LARC. Additionally, the under‐reporting of cases from private hospitals may have also skewed results. We minimised reporting bias by focusing on cases from 2018, when data entry became mandatory for colorectal surgery fellowship training centres in ANZ, which would be expected to capture the highest volume of rectal cancer cases. Furthermore, missing case data were addressed by using suitable surrogates in the registry when available. However, since the BCOR primarily records surgical cases, patients achieving cCR after NAT may not be captured, potentially leading to the reduction of cases in 2023 and an underestimation of TNT utilisation. Expanding the BCOR to include non‐surgical cases could offer a more accurate representation of treatment patterns in ANZ.

Moreover, TNT is categorised as ‘other’ NAT in the BCOR, increasing the risk of misclassification and could significantly increase the risk of bias. We aligned our classification with previous BCOR reports [19, 23] to minimise this risk. Apart from SC‐RT, LC‐CRT and TNT, the only other plausible NAT option for LARC is neoadjuvant chemotherapy alone [22], which likely remains limited in adoption across ANZ. The BCOR database also lacks the granularity necessary to analyse specific TNT regimens, such as induction or consolidation therapy [38]. Furthermore, factors such as threatened mesorectal fascia, extramural venous invasion, lateral lymph node involvement, and T and N stage subcategories, which may have influenced TNT receipt, were not included in our analysis due to limitations in the registry data dictionary. These residual confounders could affect the accuracy of our findings. Finally, the generalisability of our results may be limited, as mandatory reporting applied only to colorectal surgery fellowship centres.

## 5 Conclusion

This multinational registry study offers a snapshot of current TNT usage in surgical rectal cancer patients captured in the BCOR in ANZ and establishes a baseline for future benchmarking. It reveals a large increase in TNT utilisation for LARC, consistent with global trends despite lacking local guideline endorsement. Future research into practice patterns of specific TNT regimens and additional pre‐treatment factors influencing TNT receipt will assist clinicians, patients, and policymakers better understand the evolving landscape of LARC management.

## Author Contributions

**Ishraq Murshed:** conceptualization, methodology, investigation, validation, formal analysis, data curation, visualization, writing – original draft, writing – review and editing, project administration. **Sergei Bedrikovetski:** formal analysis, writing – review and editing. **Zachary Bunjo:** conceptualization, writing – review and editing. **Hidde M. Kroon:** software, resources, writing – review and editing. **Michelle Thomas:** conceptualization, writing – review and editing, supervision. **Tarik Sammour:** conceptualization, supervision, writing – review and editing.

## Conflicts of Interest

Associate Professor Tarik Sammour is a Speciality Editor in Colorectal Surgery for the ANZ Journal of Surgery.

## Data Availability

All data used for analysis and other material used in the study may be obtained from the corresponding author upon reasonable request.
